# Validity and Reliability of the Turkish Version of the Communicative Effectiveness Index (CETI‐TR) in Post‐Stroke Aphasia

**DOI:** 10.1002/brb3.71711

**Published:** 2026-08-20

**Authors:** Mümüne Merve Parlak, Cansu Yıldırım Tutar, Mariam Kavakci, Özlem Bizpınar Munis

**Affiliations:** ^1^ Department of Speech and Language Therapy Faculty of Health Sciences Ankara Yıldırım Beyazıt University Ankara Turkey; ^2^ Department of Speech and Language Therapy Faculty of Health Sciences İzmir Bakırçay University İzmir Turkey; ^3^ Department of Neurology Etlik City Hospital Ankara Turkey

**Keywords:** aphasia, CETI, communicative effectiveness, functional communication, stroke, validity and reliability

## Abstract

**Background:**

Assessment of communication in individuals with aphasia requires tools that capture not only linguistic abilities but also functional communication in everyday life. The Communicative Effectiveness Index (CETI) is a widely used caregiver‐reported measure; however, a validated Turkish version is not currently available.

**Objective:**

This study aimed to culturally adapt the CETI into Turkish (CETI‐TR) and to examine its validity and reliability in individuals with aphasia.

**Methods:**

This cross‐sectional study included 55 individuals with stroke (20 with aphasia, 35 without aphasia) and their caregivers. The adaptation process involved translation, back‐translation, expert review, and pilot testing. Participants were assessed using the CETI‐TR, the Language Assessment Test for Aphasia (LATA), and Quick Aphasia Battery‐Turkish Version (QAB‐TR). Internal consistency was evaluated using Cronbach's alpha. Test–retest and cross‐informant agreement (between caregivers and clinicians) were assessed using correlation analysis and intraclass correlation coefficients (ICCs). Criterion validity was examined through correlations with LATA and QAB‐TR scores, while discriminant validity was evaluated by comparing individuals with and without aphasia.

**Results:**

The CETI‐TR demonstrated excellent internal consistency (Cronbach's alpha = 0.984), with item–total correlations ranging from 0.766 to 0.964. Test–retest reliability showed high stability (ICC = 0.972). Cross‐informant agreement between caregivers and speech‐language therapists was high (ICC = 0.936), with no significant difference between ratings (*p* = 0.246). Moderate, significant correlations were found between CETI‐TR scores and LATA (*r* = 0.439, p = 0.001) and QAB‐TR (*r* = 0.524, *p* < 0.001). Individuals with aphasia had significantly lower CETI‐TR scores than those without aphasia (*p* < 0.001).

**Conclusion:**

The CETI‐TR is a valid, reliable, and clinically applicable tool for assessing functional communication in Turkish‐speaking individuals with post‐stroke aphasia. It provides an ecologically valid measure that can support clinical evaluation, intervention planning, and outcome monitoring in aphasia rehabilitation.

## 1 Introduction

Stroke remains one of the leading causes of morbidity and mortality worldwide. According to the World Stroke Organization, it is the second leading cause of death and the third leading cause of combined death and disability globally (Feigin et al. 2025). Among its many consequences, communication disorders are highly prevalent and have a profound impact on individuals’ functional independence and social participation (Baker et al. 2022; Jacobs and Ellis Jr. 2025; Winstein et al. 2016).

Aphasia is an acquired neurogenic language disorder resulting from injury to the brain—most commonly the left hemisphere—that disrupts the core components of the language network, affecting comprehension, production, reading, and writing (Sheppard and Sebastian 2021). It is one of the most common and disabling consequences of stroke, with estimates suggesting that approximately 25%–50% of stroke survivors experience aphasia in the acute phase (Engelter et al. 2006; Flowers et al. 2016; Dickey et al. 2010). The incidence varies considerably with age, ranging from roughly 15% in individuals under 65 years to over 40% in those aged 85 and older (Engelter et al. 2006). Beyond its prevalence, the long‐term prognosis is shaped primarily by initial aphasia severity, as well as lesion site and size (Hillis et al. 2018; Watila and Balarabe 2015).

Importantly, even mild aphasia can lead to significant disruptions in everyday communication, limiting individuals’ ability to engage in routine activities and maintain social relationships (Armstrong et al. 2013; Cruice et al. 2003; Poirier et al. 2024). Beyond linguistic impairment, reduced functional communication is strongly associated with adverse psychosocial outcomes, including social isolation, depression, and reduced quality of life (Hilari et al. 2010; Kauhanen et al. 2000; Berthier et al. 2023; Mohr et al. 2017). Therefore, contemporary approaches to aphasia rehabilitation emphasize not only the restoration of linguistic structures but also the enhancement of functional communication in real‐life contexts. In line with this perspective, speech‐language therapists increasingly prioritize contextually relevant and participation‐based outcomes alongside traditional impairment‐focused measures (Kagan et al. 2008; Doedens and Meteyard 2020, 2022).

A critical consideration in aphasia assessment is the perspective from which communicative effectiveness is evaluated. Clinician‐rated measures provide standardized, reproducible assessments of language structure but are typically administered in controlled clinical settings that do not reflect the complexity or variability of everyday communication (Doedens and Meteyard 2020). Self‐reported outcomes, while valuable for capturing the individual's subjective experience, may be compromised in individuals with moderate‐to‐severe aphasia due to limitations in metacognitive awareness or the linguistic demands of self‐assessment instruments (Hilari et al. 2010). In contrast, caregiver‐reported measures offer a complementary and ecologically grounded perspective, as primary communication partners observe the individual's communicative behavior across a wide range of real‐life contexts (Lomas et al. 1989; Hilari et al. 2010).

The role of communication partners in aphasia has received increasing recognition within person‐centered and social models of rehabilitation (Simmons‐Mackie et al. 2010). Communication partners are uniquely positioned to identify difficulties that may not emerge during standardized clinical assessments, including breakdowns in conversational initiation, turn‐taking, and nonverbal communication. Moreover, caregiver perspectives are essential for understanding the functional impact of aphasia on participation in family life, social relationships, and community engagement (Cruice et al. 2003, Cruice et al. 2010; Le Dorze and Brassard 1995). Incorporating caregiver‐reported outcomes into the assessment framework therefore aligns with contemporary person‐centered approaches to aphasia rehabilitation, which emphasize the importance of including the perspectives of both the individual with aphasia and their significant others in treatment planning and outcome evaluation (Chapey et al. 2000; Hersh et al. 2012).

The Communicative Effectiveness Index (CETI), developed by Lomas et al. (1989), is one such tool designed to evaluate functional communication in individuals with aphasia based on caregiver ratings. It consists of 16 items encompassing both verbal and nonverbal communication domains, rated on a 100‐point visual analog scale. The CETI provides a global index of communicative effectiveness and has been widely used to monitor functional outcomes and treatment effects (Elsner et al. 2019; Lomas et al. 1989).

To date, the CETI has been translated and culturally adapted into several languages. The Danish adaptation (Pedersen et al. 2001) reported high internal consistency (Cronbach's *α* = 0.93) and satisfactory test–retest reliability, with minimal cultural modifications required, suggesting that the instrument's core items transfer well across Northern European contexts. The Italian adaptation (Moretta et al. 2021) similarly demonstrated strong psychometric properties, including excellent internal consistency and significant correlations with impairment‐based language measures, while noting that certain items required minor linguistic revision for idiomatic clarity. The Greek adaptation (Charalambous et al. 2024) reported excellent reliability and discriminant validity, with cultural adjustments made particularly to items referencing specific social activities. The Japanese adaptation (Watanabe et al. 2011) required more substantial modifications to items involving social interaction norms, reflecting higher cultural specificity in that context. A Malayalam adaptation has also been reported (Najiya and Philip 2025). Across these studies, the CETI has demonstrated robust psychometric performance, although the degree of cultural modification required has varied depending on the linguistic and social context. Despite its widespread use, no culturally adapted and psychometrically validated Turkish version of the CETI currently exists. This represents a significant gap, given the importance of culturally sensitive assessment tools in accurately capturing communication performance within specific linguistic and social contexts. Therefore, the primary aim of this study was to develop the Turkish version of the Communicative Effectiveness Index (CETI‐TR) through cultural and linguistic adaptation, and to examine its psychometric properties, including validity and reliability, in Turkish‐speaking individuals with post‐stroke aphasia.

## 2 Methods

### 2.1 Study Design and Ethical Approval

This cross‐sectional study was conducted at the Neurology Clinic of Etlik City Hospital. Ethical approval was obtained from the Non‐Invasive Clinical Research Ethics Committee of Izmir Bakırçay University (Approval No.: 1118). All participants provided written informed consent prior to participation.

### 2.2 Participants

Participants were individuals with a confirmed diagnosis of stroke, verified by a neurologist using computed tomography (CT) or diffusion‐weighted magnetic resonance imaging (MRI). Inclusion criteria were (i) native Turkish speaker, (ii) premorbid fluency in Turkish, (iii) at least 2 weeks post‐stroke, (iv) provision of written informed consent, and (v) willingness to participate.

Exclusion criteria included (i) cognitive or language impairment due to dementia or other non‐stroke etiologies, (ii) presence of neuropsychiatric disorders, (iii) additional neurological conditions, and (iv) inability to provide informed consent independently.

Differentiation between stroke‐related language impairment and cognitive impairment attributable to concurrent vascular changes or dementia was made by the responsible neurologist based on neuroimaging findings, clinical history, and cognitive screening, with exclusion applied when cognitive impairment was judged to be the primary contributor to communication difficulties.

Aphasia diagnosis was established by a certified speech‐language therapist through comprehensive clinical evaluation using the Language Assessment Test for Aphasia (LATA) and the Quick Aphasia Battery‐Turkish Version (QAB‐TR; Toğram and Maviş 2012; Parlak and Köse 2024), standardized Turkish‐language assessment tools. Participants were classified as having aphasia when performance fell below established normative thresholds, consistent with clinical criteria confirmed jointly by the neurologist and the speech‐language therapist.

Caregivers were defined as the primary communication partner of the stroke survivor, identified as the individual who spent the most time communicating with the participant on a daily basis. Inclusion criteria for caregivers were (i) minimum daily contact of 2 h with the participant, (ii) no significant hearing or cognitive impairment that would interfere with their ability to complete the CETI‐TR, and (iii) willingness to provide written informed consent. Professional caregivers without a prior personal relationship with the participant were excluded. Prior to completing the CETI‐TR, caregivers received standardized verbal instructions from the administering researcher explaining the rating procedure and the visual analog scale format. No specific duration of relationship was required beyond the minimum 2‐week post‐stroke inclusion period, as the caregiving relationship typically commenced at or shortly after stroke onset.

**FIGURE 1:**
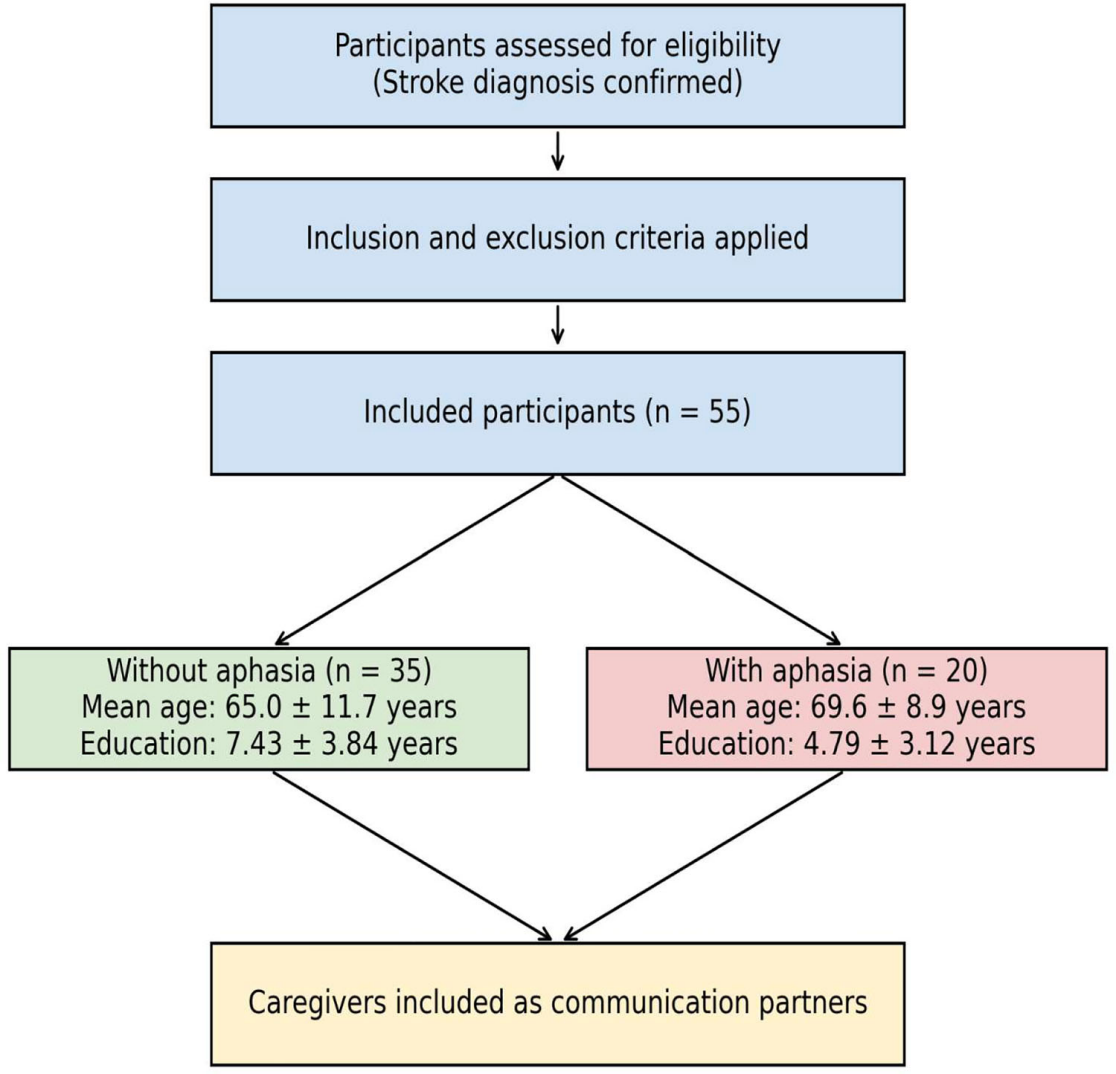
Participant selection and the demographic distribution.

A total of 55 individuals with stroke were included in the study, comprising 35 without aphasia and 20 with aphasia, along with their primary communication partners (caregivers). The mean time post‐stroke was 8.33 ± 4.87 days (median: 7). Stroke survivors without aphasia were included as a clinical reference group to enable known‐groups (discriminant) validity testing of the CETI‐TR, allowing examination of the scale's ability to distinguish individuals with impaired functional communication from those without. The mean age of participants was 66.61 ± 10.94 years, and the mean education level was 6.50 ± 3.79 years. Participants without aphasia had a mean age of 65.0 ± 11.7 years, whereas those with aphasia had a mean age of 69.6 ± 8.9 years. The mean education level was 7.43 ± 3.84 years in the non‐aphasia group and 4.79 ± 3.12 years in the aphasia group (Table 1). Although the aphasia group had significantly fewer years of formal education than the non‐aphasia group (*p* = 0.003), educational attainment was not associated with CETI‐TR total scores (*r* = −0.041, p = 0.766), indicating that education did not influence performance on the instrument. The flow of participant selection and the demographic distribution of the study sample are presented in Figure 1.

**TABLE 1:** Demographic and clinical characteristics of participants and caregivers.

Variable	No aphasia (*n* = 35)	Aphasia (*n* = 20)	All patients (*n* = 55)	Caregivers (*n* = 55)	*p*
Gender (*n*), male/female	26/9	5/15	31/24	33/22	—
Age, mean ± SD (median), years	65.0 ± 11.7 (67)	69.6 ± 8.9 (72)	66.61 ± 10.94 (69)	55.14 ± 14.84	0.085^a^
Education, mean ± SD (median), years	7.43 ± 3.84 (8)	4.79 ± 3.12 (5)	6.50 ± 3.79 (5)	7.52 ± 4.95	0.003^a^
Time post‐stroke, mean ± SD (median), days	8.89 ± 5.25 (7)	7.35 ± 4.06 (6.50)	8.33 ± 4.87 (7)	—	0.203^a^
Stroke etiology (n), Ischemic/Hemorrhagic	21/14	13/7	34/21	—	—
Relation to the patient (*n*), spouse/child/parent/other	—	—	—	30/15/6/4	—

^a^Mann–Whitney *U* test comparing the aphasia and non‐aphasia groups.

A post hoc power analysis was conducted using G*Power (version 3.1) for the discriminant validity comparison between groups (Mann–Whitney *U* test). With an observed effect size of *r* = 0.70, *α* = 0.05, and the obtained sample sizes (*n* = 35 without aphasia, *n* = 20 with aphasia), the achieved statistical power was 0.92, indicating adequate power for this specific comparison.

### 2.3 Adaptation Procedure

Permission to translate and culturally adapt the CETI (Lomas et al. 1989) was obtained from the American Speech‐Language‐Hearing Association (ASHA), to which the original copyright had been transferred. The translation and cultural adaptation process was conducted in accordance with the guidelines of Beaton et al. (2000).

The forward translation was performed by a certified professional translator whose native language is Turkish and who is fluent in English, with specific expertise in medical and healthcare terminology. The forward translator had no prior exposure to the CETI or its content before undertaking the translation.

Subsequently, the Turkish version was reviewed by an expert committee of three professionals (two speech‐language therapists and one neurologist) for conceptual and linguistic appropriateness. Each item was evaluated using predefined categories (“appropriate,” “appropriate with revision,” or “not appropriate”), and revisions were made based on expert feedback to enhance clarity and cultural relevance. During expert review, several items were identified as requiring linguistic or conceptual revision. Item 3, originally “getting someone's attention,” was retained with minimal modification, as this phrase was considered linguistically equivalent in Turkish. Item 5, “communicating feelings,” was clarified to “communicating feelings (conveying feelings)” to distinguish emotional expression from informational content, as the distinction was considered potentially ambiguous in Turkish. Item 9, originally referencing “visiting and chatting with friends and neighbors over coffee,” was adapted to “drinking tea/coffee and chatting with friends and neighbors” to reflect the cultural centrality of tea consumption in Turkish social contexts, while retaining the communicative intent of the item. Item 11, which referenced “on the telephone,” was retained but supplemented with a clarifying note specifying “using the telephone for voice communication,” as digital messaging applications are now prevalent in Turkey and could cause ambiguity. Item 14, concerning “understanding written materials,” was expanded to “understanding written content (e.g., written notes, short messages)” to enhance comprehensibility and relevance to everyday reading contexts. All modifications were reviewed by the expert panel and confirmed as preserving conceptual equivalence with the original English items.

A pilot study was then conducted with 10 stroke survivors—five with aphasia and five without aphasia—to assess comprehensibility. The pilot was carried out by the first author, a certified speech‐language therapist, in the neurology clinic of Etlik City Hospital, among individuals attending their 1‐month post‐stroke follow‐up. In individual face‐to‐face interviews, each item was presented one by one, and participants were asked whether the item was clear and understandable (yes/no). When a participant responded “no,” they were asked to suggest how the item could be reworded to improve clarity. These participant suggestions were reviewed and used to guide revisions. Based on pilot feedback, minor revisions were implemented, including minor explanatory additions to enhance item clarity (e.g., specifying “face‐to‐face” communication or “understanding written content”).

**FIGURE 2:**
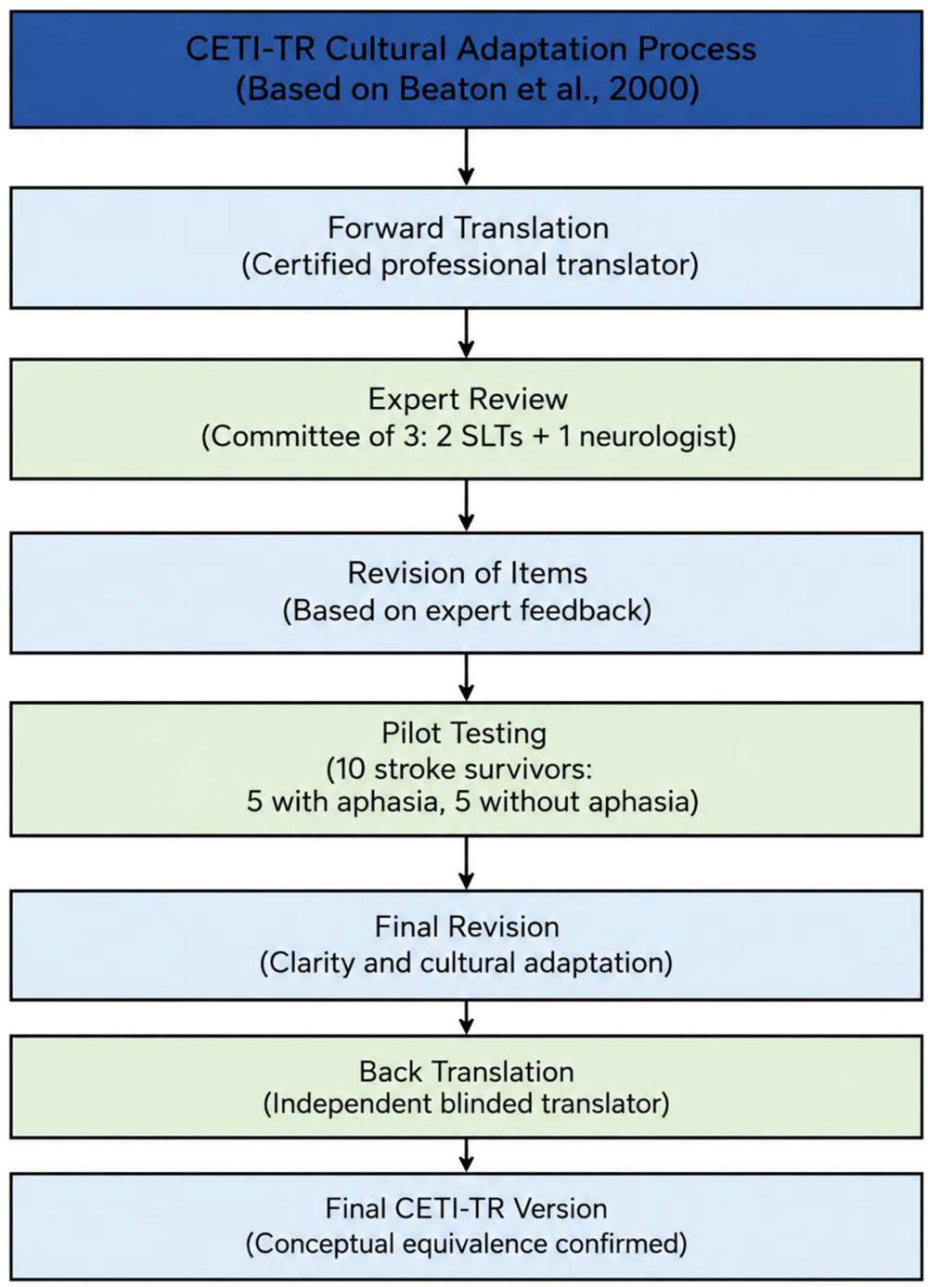
Adaptation procedure.

Finally, the revised Turkish version was back‐translated into English by an independent bilingual translator (native Turkish speaker, fluent in English) with a background in linguistics and healthcare communication, who was blinded to the original instrument. Conceptual equivalence between the original and back‐translated versions was confirmed, and the final CETI‐TR was established. The overall cultural adaptation procedure is illustrated in Figure 2.

### 2.4 Validity and Reliability Procedure

All assessments were conducted at the Neurology Clinic of Etlik City Hospital, either at the participant's bedside (for inpatients) or in a designated clinical assessment room (for outpatients), in a quiet environment to minimize distraction. Data collection was conducted in two sessions. In the first session, the LATA was administered. In the second session, participants completed the QAB‐TR, while the CETI‐TR was completed by the participants’ primary communication partners (caregivers).

Internal consistency was assessed using Cronbach's alpha coefficient. Test–retest reliability was evaluated in a subsample corresponding to approximately 20% of the total sample (*n* = 12), with the CETI‐TR re‐administered by the same caregiver after a 2‐week interval. This subsample comprised participants without aphasia, who were selected to provide a stable reference condition, since functional communication in individuals with post‐stroke acute and subacute aphasia may change over a 2‐week period due to spontaneous recovery, potentially confounding measurement instability with genuine clinical change (Hillis et al. 2018).

Agreement was examined between caregivers and speech‐language therapists—that is, between two raters with different expertise and observation contexts (home/daily life vs. clinical setting). This cross‐informant approach is consistent with the procedures used in the Italian (Moretta et al. 2021) and Greek (Charalambous et al. 2024) adaptations of the CETI. Agreement was assessed in 29 participants (9 with aphasia, 20 without) using the intraclass correlation coefficient (ICC), for whom the CETI‐TR was completed independently by both the caregiver and the speech‐language therapist on the same day.

**FIGURE 3:**
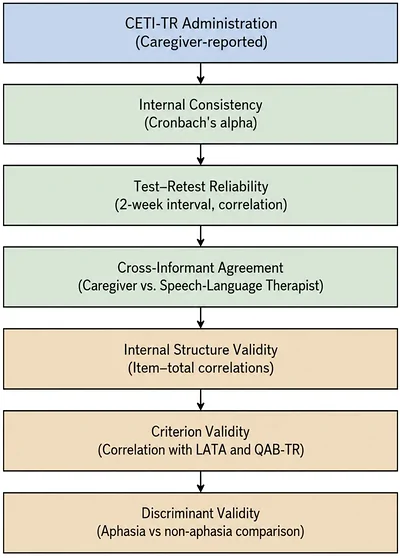
Validity and reliability procedure.

Item analysis (internal consistency assessment) was conducted through item–total correlation analyses. Criterion validity was examined by analyzing the relationships between CETI‐TR scores and scores obtained from the LATA and QAB‐TR. Discriminant validity was evaluated by comparing CETI‐TR scores between stroke survivors with and without aphasia. The sequence of validity and reliability analyses conducted in this study is summarized in Figure 3.

### 2.5 Outcome Measures

#### 2.5.1 Communicative Effectiveness Index—Turkish Version

CETI‐TR is a caregiver‐reported measure designed to assess functional communication in individuals with post‐stroke aphasia. It consists of 16 items rated on a 100‐point visual analog scale ranging from 0 (“cannot do at all”) to 100 (“can do as well as before”). The total score is calculated as the mean of all items, with higher scores indicating better functional communication.

#### 2.5.2 Language Assessment Test for Aphasia

The LATA is a standardized Turkish language assessment tool evaluating eight domains, including speech fluency, auditory comprehension, repetition, naming, reading, verbal actions, grammar, and writing (Toğram and Maviş 2012).

#### 2.5.3 Quick Aphasia Battery‐Turkish Version

QAB‐TR is a multidimensional, rapid assessment tool for language function, consisting of eight subtests, adapted from the original QAB (Wilson et al. 2018). Scores range from 0 to 10, with higher scores indicating better performance. A score of 8.825 or above is considered within normal limits (Parlak and Köse 2024).

### 2.6 Statistical Analysis

Statistical analyses were conducted using IBM SPSS Statistics (version 25.0) and Jamovi software. Descriptive statistics were presented as frequencies, percentages, means, and standard deviations. Internal consistency reliability was evaluated using Cronbach's alpha, with values ≥ 0.70 considered acceptable (Gürbüz 2019). The normality of data distribution was assessed using skewness and kurtosis values, with values within the range of ±1.5 indicating normal distribution (Tabachnick et al. 2013). Normality was additionally evaluated using the Shapiro–Wilk test.

Correlation analyses were performed using Pearson or Spearman correlation coefficients, depending on the distribution of the data. The relationship between education level and CETI‐TR scores was examined using Spearman's rank correlation coefficient. Correlation strength was interpreted as follows: very weak (< 0.20), weak (0.20–0.39), moderate (0.40–0.59), strong (0.60–0.79), and very strong (≥ 0.80) (Evans 1996). Cross‐informant agreement was evaluated using the ICC, and differences between raters were analyzed using the Mann–Whitney *U* test. Test–retest reliability was assessed using ICC, and differences between measurements were evaluated using the Wilcoxon signed‐rank test. The Mann–Whitney *U* test was used to compare age and education between the two groups (stroke survivors with and without aphasia), as well as to evaluate discriminant (known‐groups) validity by examining whether CETI‐TR scores differed between groups. A *p*‐value of < 0.05 was considered statistically significant.

## 3 Results

### 3.1 Internal Consistency and Item Analysis

The internal consistency of the CETI‐TR was found to be excellent. Corrected item–total correlation coefficients ranged from 0.766 to 0.964, indicating strong associations between each item and the overall scale score (Table 2). The Cronbach's alpha coefficient for the total scale was 0.984, demonstrating a very high level of internal consistency. Inter‐item correlation analysis further showed that all items were positively correlated with one another, with coefficients ranging from 0.589 to 0.958, reflecting moderate to very strong relationships (Table 3).

**TABLE 2:** Corrected item–total correlations and internal consistency (Cronbach's alpha) of the CETI‐TR.

Item	Corrected item–total correlation	Alpha if item deleted
Item 1	0.806	0.983
Item 2	0.845	0.983
Item 3	0.874	0.983
Item 4	0.919	0.982
Item 5	0.929	0.982
Item 6	0.869	0.983
Item 7	0.930	0.982
Item 8	0.916	0.982
Item 9	0.820	0.983
Item 10	0.919	0.982
Item 11	0.937	0.982
Item 12	0.890	0.982
Item 13	0.766	0.985
Item 14	0.964	0.981
Item 15	0.939	0.982
Item 16	0.886	0.983
Total	—	0.984

**TABLE 3:** Inter‐item and item‐total correlation matrix of the CETI‐TR.

Item	1	2	3	4	5	6	7	8	9	10	11	12	13	14	15	16	Total
**Item 1**	1.00	0.71	0.647	0.693	0.701	0.634	0.72	0.762	0.609	0.812	0.765	0.829	0.637	0.798	0.814	0.788	0.824
**Item 2**	0.71	1.00	0.78	0.798	0.864	0.913	0.789	0.752	0.744	0.804	0.793	0.765	0.70	0.874	0.798	0.761	0.858
**Item 3**	0.647	0.78	1.00	0.898	0.933	0.825	0.877	0.86	0.718	0.818	0.875	0.756	0.72	0.888	0.849	0.794	0.911
**Item 4**	0.693	0.798	0.898	1.00	0.911	0.836	0.931	0.877	0.753	0.853	0.917	0.779	0.713	0.894	0.876	0.797	0.926
**Item 5**	0.701	0.864	0.933	0.911	1.00	0.92	0.889	0.866	0.795	0.823	0.873	0.739	0.765	0.915	0.862	0.784	0.935
**Item 6**	0.634	0.913	0.825	0.836	0.92	1.00	0.851	0.791	0.743	0.795	0.815	0.708	0.701	0.892	0.792	0.756	0.881
**Item 7**	0.72	0.789	0.877	0.931	0.889	0.851	1.00	0.91	0.771	0.867	0.949	0.845	0.694	0.92	0.914	0.838	0.934
**Item 8**	0.762	0.752	0.86	0.877	0.866	0.791	0.91	1.00	0.847	0.859	0.925	0.839	0.656	0.871	0.867	0.875	0.922
**Item 9**	0.609	0.744	0.718	0.753	0.795	0.743	0.771	0.847	1.00	0.767	0.823	0.744	0.589	0.82	0.795	0.798	0.833
**Item 10**	0.812	0.804	0.818	0.853	0.823	0.795	0.867	0.859	0.767	1.00	0.878	0.936	0.786	0.884	0.869	0.903	0.938
**Item 11**	0.765	0.793	0.875	0.917	0.873	0.815	0.949	0.925	0.823	0.878	1.00	0.886	0.66	0.937	0.935	0.871	0.95
**Item 12**	0.829	0.765	0.756	0.779	0.739	0.708	0.845	0.839	0.744	0.936	0.886	1.00	0.701	0.865	0.861	0.88	0.909
**Item 13**	0.637	0.70	0.72	0.713	0.765	0.701	0.694	0.656	0.589	0.786	0.66	0.701	1.00	0.722	0.676	0.627	0.803
**Item 14**	0.798	0.874	0.888	0.894	0.915	0.892	0.92	0.871	0.82	0.884	0.937	0.865	0.722	1.00	0.958	0.869	0.971
**Item 15**	0.814	0.798	0.849	0.876	0.862	0.792	0.914	0.867	0.795	0.869	0.935	0.861	0.676	0.958	1.00	0.872	0.946
**Item 16**	0.788	0.761	0.794	0.797	0.784	0.756	0.838	0.875	0.798	0.903	0.871	0.88	0.627	0.869	0.872	1.00	0.907
**Total**	0.824	0.858	0.911	0.926	0.935	0.881	0.934	0.922	0.833	0.938	0.95	0.909	0.803	0.971	0.946	0.907	1.00

*Note*: All values presented in the table are statistically significant (*p* < 0.05).

### 3.2 Discriminant Validity

Discriminant validity analyses revealed clear differences between individuals with and without post‐stroke aphasia. The mean CETI‐TR total score reported by caregivers was 96.33 ± 4.83 in individuals without aphasia and 65.56 ± 31.96 in those with aphasia. Across all 16 items and the total score, individuals with aphasia demonstrated significantly lower functional communication performance compared to those without aphasia (*p* < 0.001) (Table 4).

**TABLE 4:** Comparison of CETI‐TR item and total scores between individuals with and without aphasia.

Item	Individuals without aphasia mean ± SD	Median	Individuals with aphasia mean ± SD	Median	*p*
1	96.86 ± 6.76	100.00	72.63 ± 30.70	80.00	< 0.001
2	93.14 ± 17.28	100.00	61.05 ± 31.78	70.00	< 0.001
3	97.71 ± 4.26	100.00	74.44 ± 33.99	90.00	< 0.001
4	96.00 ± 8.12	100.00	67.89 ± 36.14	90.00	< 0.001
5	98.00 ± 5.84	100.00	68.68 ± 37.04	80.00	< 0.001
6	94.57 ± 15.59	100.00	66.84 ± 36.22	80.00	< 0.001
7	96.57 ± 6.84	100.00	67.37 ± 35.41	80.00	< 0.001
8	98.57 ± 3.55	100.00	75.26 ± 35.33	90.00	< 0.001
9	98.43 ± 4.33	100.00	79.74 ± 29.84	90.00	< 0.001
10	95.59 ± 8.24	100.00	60.79 ± 40.15	80.00	< 0.001
11	97.43 ± 5.61	100.00	64.21 ± 36.87	80.00	< 0.001
12	96.29 ± 8.43	100.00	57.89 ± 37.50	70.00	< 0.001
13	96.86 ± 7.58	100.00	53.68 ± 43.62	70.00	< 0.001
14	96.00 ± 9.14	100.00	61.32 ± 35.82	70.00	< 0.001
15	97.43 ± 7.80	100.00	62.11 ± 35.68	70.00	< 0.001
16	94.57 ± 9.80	100.00	58.95 ± 37.25	70.00	< 0.001
Total	96.33 ± 4.83	100.00	65.56 ± 31.96	77.50	< 0.001

### 3.3 Criterion Validity

Criterion validity analyses showed that the CETI‐TR total score was moderately and significantly correlated with both the LATA total score (*r* = 0.439, *p* = 0.001) and the QAB‐TR total score (*r* = 0.524, *p* < 0.001). Significant correlations were also observed between the CETI‐TR total score and several LATA subdomains, including paragraph reading (*r* = 0.563), word–picture matching (*r* = 0.465), reading aloud and following instructions (*r* = 0.572), picture naming (*r* = 0.453), word reading (*r* = 0.426), and automatic speech (*r* = 0.407). Similarly, within the QAB‐TR subdomains, the significant correlations were identified in word finding (*r* = 0.579), word comprehension (*r* = 0.455), and grammar (*r* = 0.422), all of which were statistically significant (Table 5).

**TABLE 5:** Correlations between CETI‐TR total score and LATA and QAB‐TR scores and subdomains.

	Variable	*r*	*p*
LATA	Automatic speech	0.407	0.002
	Total speech fluency score	0.396	0.003
	Understanding commands	0.317	0.019
	Sentence variety comprehension	0.449	0.001
	Total auditory comprehension	0.360	0.007
	Categorical naming	0.285	0.037
	Picture naming	0.453	0.001
	Total naming score	0.406	0.002
	Reading aloud and following instructions	0.572	< 0.001
	Letter/number reading	0.282	0.039
	Word reading	0.426	0.001
	Word–picture matching	0.465	< 0.001
	Paragraph reading	0.563	< 0.001
	Total reading score	0.373	0.006
	Total grammar score	0.370	0.006
	Total verbal actions score	0.297	0.029
	LATA total	0.439	0.001
QAB‐TR	Word comprehension	0.455	0.001
	Sentence comprehension	0.398	0.003
	Word finding	0.579	< 0.001
	Grammar	0.422	0.002
	Motor speech	0.317	0.022
	Reading	0.371	0.007
	QAB‐TR total	0.524	< 0.001

Abbreviations: LATA, the Language Assessment Test for Aphasia; QAB‐TR, Quick Aphasia Battery‐Turkish Version.

### 3.4 Cross‐Informant Agreement

The mean CETI‐TR total score was 82.88 ± 20.96 (median: 90.63) for speech‐language therapists and 86.14 ± 20.92 (median: 93.13) for caregivers. No statistically significant difference was observed between the two raters (*p* = 0.246). The ICC indicated a high level of agreement between raters (ICC = 0.936, 95% CI: 0.88–0.97).

### 3.5 Test–Retest Reliability

Based on the test–retest analysis of 12 individuals without aphasia, the mean CETI‐TR total score was 96.36 ± 3.74 at the first measurement and 96.88 ± 3.45 at the second measurement, with median values of 99.38 and 100.00, respectively. No statistically significant difference was observed between the two measurements (*p* = 0.600). The ICC indicated excellent test–retest reliability (ICC = 0.972).

## 4 Discussion

This study provides the first cultural and linguistic adaptation of the CETI‐TR and demonstrates that it is a reliable and clinically applicable tool with promising validity evidence for assessing functional communication in individuals with post‐stroke aphasia. By capturing communication performance in everyday contexts through caregiver ratings, the CETI‐TR addresses an important gap in aphasia assessment that is not fully covered by impairment‐based language measures. These findings are particularly relevant given the growing recognition that communication assessment in aphasia should extend beyond linguistic accuracy to include functional communication in daily life (Brandenburg et al. 2017; Doedens and Meteyard 2022).

The CETI‐TR is well suited for integration into routine clinical practice in neurology, neurorehabilitation, and speech‐language therapy settings in Turkey. Its brief administration time (approximately 5–10 min for caregiver completion) and straightforward visual analog scale format make it feasible for use even in acute and subacute care contexts where time constraints are significant. As a caregiver‐reported measure, the CETI‐TR can be completed independently by the primary communication partner without requiring direct participation of the individual with aphasia, which is particularly advantageous in cases of severe aphasia where self‐report is not feasible. The CETI‐TR is best understood as a complement to, rather than a replacement for, impairment‐based language assessments such as the LATA or QAB‐TR. While impairment‐based tools provide detailed profiles of linguistic strengths and weaknesses, the CETI‐TR captures the translation of these abilities into functional communication in everyday life—a distinction that is clinically critical for rehabilitation planning and goal‐setting. Clinicians may use CETI‐TR scores to identify discrepancies between linguistic ability and functional communication performance, which may indicate the presence of communicative participation restrictions not captured by standard language tests. In rehabilitation settings, the CETI‐TR may be administered at baseline, at regular intervals during treatment, and at discharge to monitor changes in functional communication over time. Although the present cross‐sectional design did not permit assessment of responsiveness, the scale's demonstrated ability to discriminate between individuals with and without post‐stroke aphasia suggests potential utility as an outcome measure in intervention studies—a possibility that should be confirmed through future longitudinal research. Serial CETI‐TR assessments may also facilitate meaningful conversations between clinicians, individuals with aphasia, and their families about treatment goals and progress, thereby supporting shared decision‐making and person‐centered care.

The CETI‐TR demonstrated excellent internal consistency, as reflected by a Cronbach's alpha coefficient of 0.984 and high item–total correlations ranging from 0.766 to 0.964. These findings indicate that all items consistently measure the same underlying construct of functional communication and that the scale has a highly homogeneous structure. According to established criteria in scale development, item–total correlations above 0.30 are considered acceptable (Büyüköztürk 2018); therefore, the substantially higher values observed in this study provide strong evidence for the internal coherence of the scale. These results in individuals with post‐stroke aphasia align with findings reported in previous CETI adaptation studies, supporting the robustness of the scale across different cultural contexts.

The CETI‐TR also demonstrated strong reliability across time and informants. Test–retest analysis revealed no significant differences between repeated measurements and showed a very high correlation between scores, indicating high temporal stability. Similarly, cross‐informant agreement analysis demonstrated excellent agreement between caregivers and speech‐language therapists, suggesting that the scale provides consistent results across different informants. These findings are consistent with previous CETI studies and indicate that the CETI‐TR is a stable and informant‐independent measurement tool (Fucetola and Tabor Connor 2015; Lomas et al. 1989). Comparing caregiver and clinician ratings has become an established practice in recent CETI adaptation research (Moretta et al. 2021; Charalambous et al. 2024). The high agreement observed in the present study (ICC = 0.936) is consistent with these adaptations and supports the applicability of the CETI‐TR by non‐professional caregivers. This is particularly valuable for continuity of care, as caregiver reports can bridge periods when the treating clinician changes. Notably, Moretta et al. (2021) reported that caregivers tended to rate communicative abilities slightly more positively than clinicians, a pattern mirrored by the small, non‐significant difference observed here (86.14 vs. 82.88). It should be emphasized, however, that this analysis reflects agreement between informants with different expertise and observation contexts rather than classical inter‐rater reliability between two raters with the same expertise.

Criterion validity was supported by moderate, statistically significant correlations between CETI‐TR scores and established language assessment tools, including LATA and QAB‐TR. These findings align with the expectation that functional communication is associated with language performance while also reflecting that these constructs are not identical. This distinction is consistent with previous research demonstrating that improvements in linguistic abilities do not always directly translate into improvements in functional communication (Brandenburg et al. 2017; Rofes et al. 2015). Therefore, the CETI‐TR appears to capture complementary and clinically meaningful aspects of communication that extend beyond traditional impairment‐based measures. Although the moderate correlations obtained are consistent with expectations and with findings from previous CETI adaptation studies, the use of impairment‐based measures as criterion standards represents a limitation from a convergent validity perspective, as the CETI‐TR is designed to measure functional communication rather than linguistic structure per se. Stronger criterion validity evidence could be obtained through comparison with validated functional communication measures and participation‐focused or quality‐of‐life instruments. Specifically, the Turkish adaptations of the Aphasia Impact Questionnaire‐21 (AIQ‐21‐TR; Yaşar et al. 2022) and the Stroke and Aphasia Quality of Life Scale‐39 (SAQOL‐39‐TR; Noyan‐Erbaş and Toğram 2016) represent appropriate criterion measures for future validation studies. These measures capture the communicative and psychosocial dimensions of aphasia that are most closely aligned with the construct of functional communication effectiveness assessed by the CETI‐TR.

The ability of the CETI‐TR to discriminate between individuals with and without aphasia provides further support for its known‐groups (discriminant) validity. Individuals with aphasia demonstrated significantly lower scores across all items and the total score compared to those without aphasia, reflecting the broad impact of aphasia on both verbal and nonverbal communication abilities. These findings are consistent with previous studies and highlight the sensitivity of the CETI as a measure of functional communication impairment (Charalambous et al. 2024; Lomas et al. 1989). Although the aphasia group had significantly fewer years of formal education than the non‐aphasia group, education was not associated with CETI‐TR scores, suggesting that the between‐group differences reflect the presence of aphasia rather than educational attainment.

The psychometric properties of the CETI‐TR compare favorably with those reported in previous CETI adaptation studies. With respect to internal consistency, the Cronbach's alpha of 0.984 obtained in the present study is comparable to values reported in the original English version (high internal consistency; Lomas et al. 1989), the Danish adaptation (*α* = 0.96; Pedersen et al. 2001), the Italian adaptation (caregiver *α* = 0.92; speech therapist *α* = 0.97; Moretta et al. 2021), the Greek adaptation (*α* > 0.95; Charalambous et al. 2024), and the Malayalam adaptation (*α* = 0.98; Najiya and Philip 2025), indicating consistently high internal coherence across cultural contexts. Regarding test–retest reliability, the ICC of 0.972 observed in the present study aligns with the excellent stability reported in the Malayalam adaptation (ICC = 0.99; Najiya and Philip 2025) and is numerically higher than the values reported for the Greek adaptation (ICC = 0.88; Charalambous et al. 2024), the Italian adaptation (Spearman's *ρ* = 0.93 for caregivers and 0.84 for speech therapists; Moretta et al. 2021), and the original Danish adaptation (*r* = 0.86; Pedersen et al. 2001), although direct comparison is limited by differences in sample composition, statistical method, and retest interval. With respect to caregiver–clinician (cross‐informant) agreement, the high concordance observed in the present study (ICC = 0.936) is consistent with the cross‐informant findings reported in the Italian (Moretta et al. 2021) and Greek (Charalambous et al. 2024) adaptations. Notably, Moretta et al. (2021) were the first to compare CETI ratings provided by raters of differing expertise (speech therapists versus caregivers) and explicitly noted that such a comparison constitutes an indicator of the scale's applicability by non‐professional caregivers rather than a substitute for classical inter‐rater reliability—an interpretation directly aligned with the present study. Discriminant validity, demonstrated by significantly lower CETI‐TR scores in individuals with post‐stroke aphasia, replicates findings reported across prior adaptations. Overall, the CETI‐TR demonstrates a psychometric profile that is highly congruent with the existing cross‐cultural validation literature, supporting its robustness as a measure of functional communication across diverse linguistic and cultural contexts.

### 4.1 Limitations

Despite its strengths, this study has several limitations that should be considered when interpreting the findings. First, the sample size was relatively small, which may limit the generalizability of the results and may have influenced the stability of the reliability estimates, particularly the very high ICCs observed. In particular, the test–retest reliability was based on a small subsample (*n* = 12) drawn from non‐aphasic participants and assessed over a 2‐week interval; although this subsample yielded excellent temporal stability (ICC = 0.972), this estimate should be interpreted with caution and confirmed in larger samples. Notably, test–retest data were not obtained for individuals with chronic aphasia, and the temporal stability of the CETI‐TR in this population therefore remains to be established. Second, the cross‐informant analysis comparing caregiver and clinician ratings should be regarded as an indicator of the scale's applicability by non‐professional caregivers rather than as a substitute for classical inter‐rater reliability, consistent with the interpretation proposed by Moretta et al. (2021). Third, the cross‐sectional design precludes conclusions regarding the responsiveness of the CETI‐TR to changes over time or to intervention effects. Finally, due to the relatively small sample size, factor analysis could not be conducted to examine the underlying structure of the scale.

### 4.2 Future Directions

Several directions for future research are indicated by the findings and limitations of the present study. First, validation studies with larger samples, including substantially larger aphasia subsamples with greater severity diversity, are needed to enable factor analysis and to generate more stable and generalizable reliability and validity estimates. Second, test–retest reliability should be specifically evaluated in individuals with post‐stroke aphasia across a range of severity levels and at multiple time points in the recovery trajectory. Third, criterion validity should be examined using functional communication measures (e.g., AIQ‐21‐TR) and quality‐of‐life instruments (e.g., SAQOL‐39‐TR) to provide a more ecologically valid validity framework. Fourth, responsiveness to change following speech‐language therapy intervention should be evaluated using longitudinal designs to determine whether the CETI‐TR is sensitive to clinically meaningful changes in functional communication. Fifth, content validity should be formally assessed following COSMIN methodology, incorporating the perspectives of individuals with aphasia and their caregivers. Finally, normative data for the CETI‐TR should be developed from large, representative Turkish samples to support the clinical interpretation of individual scores.

## 5 Conclusion

In conclusion, the present findings indicate that the CETI‐TR is a valid and reliable instrument for assessing functional communication in Turkish‐speaking individuals with post‐stroke aphasia, demonstrating excellent internal consistency, strong test–retest reliability, and high caregiver–clinician agreement. As a clinically useful and informant‐independent tool, the CETI‐TR may be readily incorporated into routine clinical and rehabilitation practice. Its use may contribute to a more comprehensive evaluation of communication abilities and support both clinical decision‐making and research in aphasia rehabilitation.

## Author Contributions

**Mümüne Merve Parlak**: conceptualization, investigation, methodology, visualization, validation, data curation, writing – review and editing, software. **Cansu Yıldırım**: data curation, writing – original draft, visualization, formal analysis, software. **Mariam Kavakci**: methodology, conceptualization, funding acquisition, writing – review and editing, supervision. **Özlem Bizpınar Munis**: conceptualization, methodology, software, supervision, project administration.

## Funding

The authors have nothing to report.

## Ethics Statement

All procedures performed in studies involving human participants were in accordance with the ethical standards of the institutional and/or national research committee and with the 1964 Helsinki Declaration and its later amendments or comparable ethical standards. Before the recruitment, written informed consent was obtained from each participant and their respective caregiver. Ethical approval was granted by the Non‐Invasive Clinical Research Ethics Committee of Izmir Bakırçay University (Approval No.: 1118).

## Conflicts of Interest

The authors declare no conflicts of interest.

## Data Availability

All data generated or analyzed during this study are included in this article. Further inquiries can be directed to the corresponding author.
